# Exploiting the molecular diversity of the synapse to investigate neuronal communication: A guide through the current toolkit

**DOI:** 10.1111/ejn.15848

**Published:** 2022-11-04

**Authors:** Jacopo Lamanna, Mattia Ferro, Sara Spadini, Antonio Malgaroli

**Affiliations:** ^1^ Center for Behavioral Neuroscience and Communication (BNC) Vita‐Salute San Raffaele University Milan Italy; ^2^ Faculty of Psychology Vita‐Salute San Raffaele University Milan Italy; ^3^ Department of Psychology Sigmund Freud University Milan Italy; ^4^ San Raffaele Turro, IRCCS Ospedale San Raffaele Milan Italy

**Keywords:** endo‐exocytosis, genetically encoded, indicator, optogenetics, synaptic transmission

## Abstract

Chemical synapses are tiny and overcrowded environments, deeply embedded inside brain tissue and enriched with thousands of protein species. Many efforts have been devoted to developing custom approaches for evaluating and modifying synaptic activity. Most of these methods are based on the engineering of one or more synaptic protein scaffolds used to target active moieties to the synaptic compartment or to manipulate synaptic functioning. In this review, we summarize the most recent methodological advances and provide a description of the involved proteins as well as the operation principle. Furthermore, we highlight their advantages and limitations in relation to studies of synaptic transmission in vitro and in vivo. Concerning the labelling methods, the most important challenge is how to extend the available approaches to the in vivo setting. On the other hand, for those methods that allow manipulation of synaptic function, this limit has been overcome using optogenetic approaches that can be more easily applied to the living brain. Finally, future applications of these methods to neuroscience, as well as new potential routes for development, are discussed.

## Graphical abstract

Chemical synapses are tiny and overcrowded environments enriched with thousands of protein species. Many efforts have been devoted to developing sensors and actuators able to evaluate and control synaptic communication. Most of these recent tools are based on the engineering of one or more specific synaptic proteins used to target active moieties to the synaptic compartment. In this review, we summarize the most recent methodological advances in this field and provide a description of the involved proteins as well as the operation principle.

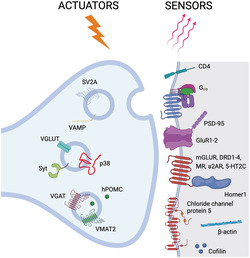

**Abbreviations**
AMPA: alpha‐amino‐3‐hydroxy‐5‐methyl‐4‐isoxazole‐propionic acid
Arch3: archaerhodopsin‐3
AZs: active zones
CALI: chromophore‐assisted light inactivation
ChR2: channelrohdopsin‐2
CiVSP: Ciona intestinal VSP
CNO: clozapine‐N‐oxide
CNS: central nervous system
CRY2: cryptochrome 2
CTZ: coelenterazine
DA: dopamine
DREADDs: Designer Receptors Exclusively Activated by Designer Drug
DTE: dendritic targeting element
FFNs: fluorescent false neurotransmitters
GABA: gamma‐aminobutyric acid
GECIs: genetically encoded calcium indicators
GEVIs: genetically encoded voltage indicators
GFP: green fluorescent protein
GPCRs: G‐coupled receptors
GRASP: GFP Reconstitution Across Synaptic Partners
IL‐2R: interleukin‐2 receptor
ITet: immuno‐tetanus toxin
LOV: light‐oxygen‐voltage
LTP: long‐term potentiation
mGluR2: metabotropic glutamate receptor 2
miniSOG: mini singlet oxygen generator
PBP: periplasmic binding protein
POO: parapinopsin
PSD: postsynaptic density
RIM‐BPs: RIM‐binding proteins
RIM: Rab3‐interacting molecule
SB: Synbond
SEP: Super Ecliptic pHluorin
SM: Sec1/Munc18‐like
SN: SuperNova
SNARE: soluble *N*‐ethylmaleimide‐sensitive factor attachment protein receptor
syb‐2: synaptobrevin‐2
syp: synaptophysin
syt: synaptotagmin
VDCCs: voltage‐dependent calcium channels
VGAT: vesicular GABA transporter
VGCCs: voltage‐gated calcium channels
VMAT2: vesicular monoamine transporter 2
VS: voltage sensor
VSP: voltage sensitive phosphatase

## 1 INTRODUCTION

Synapses represent the unique morphological and functional neuronal compartment specialized in communication. Due to its limited size and high density of molecular elements and organelles, the functional study of the synapse has been representing a challenge since the very beginning of neurophysiology (Fatt & Katz, 1952). As usual in science, most of the recent advances in understanding synaptic functioning can be ascribed to specific technological and methodological improvements. In the last decades, novel methods of microscopy, molecular biology, genetics and biochemistry were developed to directly sense or interfere with synaptic transmission and plasticity. An example is the development of super‐resolution fluorescence microscopy (Nosov et al., 2020), which broke the diffraction limit allowing the visualization of sub‐synaptic compartments, such as synaptic vesicles, which can be followed in their motion and fusion dynamics (Westphal et al., 2008), and single receptor proteins that traffic on the plasmatic membrane (Groc & Choquet, 2020).

Neuronal communication can be investigated using several approaches that allow the elicitation and detection of electrochemical signals at non‐synaptic compartments, including the soma, e.g. by evoking action potentials using optogenetics and measuring electrical activation or calcium rises at the target brain region. Nevertheless, the most specific approaches allow to focus on the main site of neuronal communication, which is the synapse, in a manner that is as much as possible separated from the electrical activity of neurons. This possibility is of crucial importance especially when investigating synaptic plasticity phenomena, which are known to regulate the efficacy of communication and to occur primarily, if not solely, at the synaptic level.

Synapses are enriched with thousands of protein species, of which more than 400 different isoforms are found in synaptic vesicles and more than 1000 are found at the postsynaptic density (PSD) (O'Rourke et al., 2012). These numbers testify a huge molecular diversity, which likely confers high heterogeneity to synaptic molecular profiles, but clearly, only a fraction of protein types is unique of synaptic compartments. These include the SNARE (soluble *N*‐ethylmaleimide‐sensitive factor attachment protein receptor) and SM (Sec1/Munc18‐like) proteins, which are essential for the process of vesicle fusion, which occurs at active zones (AZs) and supports the release of neurotransmitters during synaptic transmission. In the presynaptic AZs, together with SNAREs/SMs and voltage‐gated calcium channels (VGCCs), several other proteins are present, including Rab3‐interacting molecule (RIM) and RIM‐binding proteins (RIM‐BPs). RIM interacts with vesicular Rab3 for vesicle docking and with Munc13 for vesicle priming but is also responsible for the clustering of VGCCs at AZs, which grants calcium‐dependent exocytosis (Südhof, 2013). Furthermore, the expression of neurotransmitter receptors and transporters, both in the pre‐ and postsynaptic compartments, strongly diversifies synapses in their physiological profile.

Such molecular diversity represents a valuable resource for the development of novel tools that allow to investigate synaptic functioning. Indeed, several synaptic proteins have been already exploited to create sensors and actuators proved effective for measuring and modulating synaptic function. This review outlines and discusses the available toolkit and provides a selection based on the successful usage of specific synaptic proteins.

## 2 EXPLOITING SYNAPTIC PROTEINS TO MEASURE SYNAPTIC COMMUNICATION

The basic engineering of synaptic proteins was originally aimed to localize and quantify their amount within the neuron, to obtain morphological information about the structural modifications of different neuronal compartments. This objective was easily achieved by the fusion with fluorescent proteins of specific synaptic proteins (i.e., *tagging*), either presynaptic (D'Acunzo et al., 2014; Miesenböck et al., 1998; Nonet, 1999) or postsynaptic (Ebihara et al., 2003), and through more advanced approaches. For instance, Shu and colleagues exploited the genetically encoded singlet oxygen generator (miniSOG) (for review, see Souslova et al., 2017) to visualize pre‐ and postsynaptic proteins (SynCAM1 e SynCAM2, respectively) by the integrated use of fluorescence and electron microscopy (Shu et al., 2011).

### 2.1 Fluorescence‐based sensors of synaptic vesicle pH

Several endogenous synaptic proteins have also been exploited to sense synaptic activity at different locations. One of the first examples of such approach is represented by the intraluminal pH sensor synapto‐pHluorin (Miesenböck et al., 1998; Sankaranarayanan & Ryan, 2000). Synapto‐pHLuorin is a genetically encoded presynaptic vesicle recycling sensor. The sensor was initially based on a pH‐sensitive (ecliptic) form of green fluorescent protein (GFP) fused to the vesicle membrane protein synaptobrevin‐2 (VAMP‐2, syb‐2). Inside synaptic vesicles, at the acid pH level, synapto‐pHluorin is non‐fluorescent (quenched). When vesicular exocytosis occurs, synapto‐pHluorin is exposed to the extracellular space (physiological pH) and emits the fluorescent signal. Different fluorescent proteins have higher or lower pKa levels (e.g., the acidic dissociation constant of YFP is higher than the one of GFP). Following endocytosis, vesicles become re‐acidified, and the cycle can start again. Later evolution of this method includes fusion of pHluorin to different presynaptic proteins, such as the synaptic vesicle transmembrane protein synaptophysin for sypHy (Granseth et al., 2006) and pHoenix (Rost et al., 2015), the glutamate transporter VGLUT1 (Balaji & Ryan, 2007; Voglmaier et al., 2006), all synaptotagmin (syt, p65) isoforms (Dean et al., 2009; Dean, Dunning, et al., 2012) and VAMP‐4 (Li, 2011). Notably, although these versions of the sensor only required to change the native synaptic fusion protein, both the sensitivity and the specificity of the tool were improved, helping to answer relevant questions about the role and functioning of these SNARE proteins as well as of the associated organelles. Also red‐shifted exocytosis sensors based on the same approach were developed, with mOrange and mOrange2 fused to either VAMP‐2 (syb2‐mOrange) (Ramirez et al., 2012) or VGLUT1 (VGLUT1‐mOr2) (Li, 2011), respectively. Similar to the ecliptic GFP, mOrange/mOrange‐2 fluorescence appears to be quenched by the low intra‐vesicle pH and restored upon vesicle fusion. Further red‐shifted variants were obtained by fusing pHTomato to synaptophysin (p38, syp) (sypHTomato) (Li & Tsien, 2012) and, recently, pHmScarlet to VAMP‐2 (Liu et al., 2021). Furthermore, N‐terminal pHluorin‐tagged mGluR7 was deployed in neurons as an independent assay of receptor internalization, allowing live imaging of surface receptors in real time (Pelkey et al., 2007). Finally, localization of pHluorin in synaptic vesicles was also achieved by fusing it to the C‐terminus of the vesicular gamma‐aminobutyric acid (GABA) transporter (VGAT) (Santos et al., 2013), as well as by placing it into an intraluminal loop of the synaptic vesicular protein 2A (Zhang et al., 2015). A major limitation of such tools is that they do not retain the signal, which is transient, so that complex imaging techniques must be applied to obtain the readout in vivo. Another limitation is related to the signal to noise ratio, which is likely not sufficient to obtain synaptic activity mapping for large regions of the living brain with single synapse resolution. Finally, the endocytosis activity informs only about presynaptic activation and does not distinguish between inhibitory and excitatory forms of transmission.

### 2.2 Staining fusing vesicles for the activity labeling of synapses

Up to the 90s, it was impossible to visualize brain synapses in living neurons. The only exception was represented by catecholaminergic boutons, due to the strong autofluorescence of the catechol group (Eränkö, 1978). Clearly, since the initial purification and cloning of synaptic vesicle proteins, it became possible to label synapses by immunostaining, albeit limited to fixed cells and tissues. In the 90s, the idea that presynaptic boutons could be visualized through cycles of exo‐endocytosis emerged and a few techniques were developed (Betz & Bewick, 1993; Malgaroli et al., 1995). With FM dyes, the signal was transient, thus requiring live imaging of the destaining process (Betz & Bewick, 1993), while using anti‐synaptotagmin, it was possible to image both the uptake dynamics and the final cumulative signal as a readout of neurotransmitter release rate (Malgaroli et al., 1995). Unfortunately, those methods were effective only in vitro.

After two decades, a similar idea was used to develop a genetically encoded probe (Ferro et al., 2017). The sensor, named SynaptoZip, exploited an engineered dimeric system named *Velcro*. This system is composed of two peptides, named ZIP and Synbond (SB), which can interact forming a coiled‐coil heterodimer with high affinity and stability (O'Shea et al., 1993). ZIP was fused to the intraluminal end of VAMP‐2 (N‐terminus), whereas eGFP was fused to its C‐terminus for localization purposes (eGFP‐SynaptoZip). Furthermore, the SB peptide, with a molecular weight of 3.9 KDa, was fluorescently tagged with Alexa Fluor dyes and delivered to the neural tissue: The idea is that when vesicles fuse to the presynaptic membrane upon exocytosis, the intraluminal ZIP moiety is exposed to the synaptic cleft as a bait and stably binds SB. Hence, SynaptoZip sensors can accumulate SB molecules into vesicles within each round of exo‐endocytosis and retain them for later quantification, thus generating a cumulative, activity‐dependent staining of synaptic boutons. SynaptoZip was shown to reliably detect and quantitatively report presynaptic activation with single synapse resolution both in vitro and in vivo (Ferro et al., 2017). In addition, in a recent study, eGFP‐SynaptoZip and its red‐shifted variant mCherry‐SynaptoZip were used in the same animal to compare the activity at two synaptic pathways originating from the prelimbic and infralimbic portions of the prefrontal cortex and converging to the basolateral amygdala, where SB was delivered (Lamanna et al., 2022). Such approach provides a ratiometric measure of synaptic activity that further improves the reliability of the method as SB reaches the two populations of synapses with the same concentration and timing. A limitation of this method, besides its purely presynaptic nature, relates to the need for external delivery of SB, which would benefit from the development of specific equipment, as this requirement can limit the temporal dynamics of activity labelling.

### 2.3 Detecting synaptic functional connectivity

Another tool to sense synaptic communication is based on the GFP Reconstitution Across Synaptic Partners (GRASP) (Feinberg et al., 2008). The GRASP approach was initially proposed as a tool to label synaptic contacts: two separate fragments of GFP (‘split’ GFP), named spGFP1‐10 and spGFP11, were expressed on pre‐ and post‐ synaptic membranes, respectively, by fusing them to different membrane proteins (PTP‐3A, CD4, Nlgn1). The authors showed that GFP could be successfully reconstituted trans‐synaptically in defined populations of synaptic contacts. Advanced versions of the same approach were later developed based on different associations with neurexin and neuroligin isoforms (Kim et al., 2012; Shearin et al., 2018; Tsetsenis et al., 2014; Yamagata & Sanes, 2012). These GRASP methods can provide valuable information about the presence of putatively functional synaptic contacts but cannot sense synaptic activity. Nevertheless, in a recent evolution of GRASP (syb‐GRASP) (Macpherson et al., 2015), VAMP‐2 was selected as the presynaptic protein to be fused to the first split‐GFP fragment, so that GFP reconstitution became dependent upon vesicle fusion and exposition of the spGFP1‐10 fragment to the synaptic cleft, where the second fragment can be found (spGFP11 fused to CD4) (Macpherson et al., 2015). Multi‐colour versions have also been developed allowing to distinguish different synaptic circuits: X‐RASP (Macpherson et al., 2015) and CRASP (Li et al., 2016). Nevertheless, activity dependence of syb‐GRASP has not been sufficiently characterized, and its application to address specific questions about neuronal communications is still missing. This is likely due to two major weaknesses: (i) It is not known to which extent and how long the presynaptic moieties of the syb‐GRASP system are sequestered after fusion and GFP reconstitution; (ii) such sequestration applies also to the involved vesicles, affecting the following transmission capability of the synapse. As the matter of fact, activity dependence of syb‐GRASP appears only as a feature able to enhance specificity of synaptic connectivity analysis and not to provide a functional readout; in addition, such improvement might bring important side effects, including a reduced signal intensity and the underestimation of connectivity strength (Shearin et al., 2018).

### 2.4 Genetically encoded calcium indicators (GECIs) brought at the synapse

A totally different approach to sense synaptic activation was based on GECIs (see Lin & Schnitzer, 2016; Rose et al., 2014 for review), a large family of engineered proteins exhibiting changes in fluorescence upon binding to Ca^2+^. The GCaMP family is the largest GECI family and consists of a circularly permuted GFP fused, at the C‐terminus, to calmodulin (CaM) and, at the N‐terminus, to the M13 fragment of myosin light chain kinase, a target sequence of calmodulin. When calcium binds to CaM, a conformational change in GFP occurs, due to calcium‐CaM‐M13 interaction, thus changing the fluorescence intensity (Nakai et al., 2001). This probe, particularly useful to visualize the levels of intracellular calcium in living cells, has high affinity to calcium, and dissociation kinetics are independent from its concentration. GCaMP‐family GECIs underwent many improvements through mutagenesis and selection, for example by reducing the half‐decay timing, by changing the spectral properties and by reaching an exceptional signal to noise ratio. Valid attempts showed that, even with neuron‐wide cytosolic expression of these calcium sensors, imaging the synaptic terminal can be feasible for ascertaining their specific physiological role in vivo (Sun et al., 2016). Nevertheless, more specific information about Ca^2+^ dynamics of presynaptic activation was obtained when GECI expression was localized at the presynaptic compartment, by fusing GCaMP‐2 to the cytoplasmic end of synaptophysin: The resulting probe was proved functional in vivo in the zebrafish (Dreosti et al., 2009). Further evolutions of this approach include red‐shifted GECIs (e.g., RCaMP and R‐GECO1, Akerboom et al., 2013; Zhao et al., 2011), as well as the fusion of synaptophysin to GCaMP‐3 (SyGCaMP3) (Li, 2011), which was successfully applied in vitro together with the VGLUT1‐mOr2 exocytosis sensor, aiming at characterizing the relationship between presynaptic calcium rises and vesicle fusion. Similar tools were later developed using GCaMP5G (SyGCaMP5G) (Akerboom et al., 2012) and GCaMP6 (SyGCaMP6) (Mahn et al., 2016), both associated with synaptophysin. A further development of the GECI system is represented by the construct sypHy‐RGECO, resulting from the fusion of sypHy (Granseth et al., 2006) with the red‐shifted calcium indicator R‐GECO1 (Jackson & Burrone, 2016). The resulting reporter, successfully applied in vitro, concurrently images presynaptic calcium influx and vesicle exocytosis in the same presynaptic terminal. GECIs have been also targeted to the postsynaptic compartment in order to detect synaptic‐evoked Ca^2+^ transient in spines, by fusing GCaMP2 to β‐actin (GCaMP2‐actin) and PSD‐95 (PSD95‐GCaMP2) (Mao et al., 2008). The latter was also fused to GCamp5K in a later application (PSD‐95‐GCamp5K) (Leitz & Kavalali, 2014). Finally, a recent approach based on a GECIs was recently provided by Perez‐Alvarez and colleagues (Perez‐Alvarez et al., 2020): The authors used CaMPARI, a photoconvertible calcium sensor (Fosque et al., 2015) fused to synaptophysin, to get permanent labelling of presynaptic activation (more precisely, the authors used the second version of the sensor, CaMPARI‐2, Moeyaert et al., 2018). Approaches based on GECIs represent some of the most effective ones in this field; nevertheless, as for any other sensor discussed here, they can only provide the readout of a specific step of synaptic transmission, the presynaptic calcium rise. This event occurs very early in the transmission process and is hardly distinguished from the electrical activity of the neuron (or axon): This can translate to an important limitation for those who want to investigate the link between presynaptic electrical activity and either neurotransmitter release or postsynaptic activation. Indeed, because reliability of the fusion machinery is far from absolute, especially in the central nervous system (CNS), the real relationship between voltage and calcium rises in the presynaptic terminal and the following neurotransmitter release remains unknown. In addition, the stochastic nature of quantal transmission, not captured by these sensors, is regarded as a key aspect for information processing in the brain (Abenavoli et al., 2000; Lamanna et al., 2015; Ribrault et al., 2011).

### 2.5 Using membrane voltage indicators for measuring membrane depolarization at the synapse

Although most genetically encoded voltage indicators (GEVIs) and voltage sensor (VS) dyes are not engineered with specific synaptic proteins and thus not central to the aim of our dissertation, they undoubtedly provide an effective tool to measure postsynaptic membrane depolarization. A recent review (Panzera & Hoppa, 2019) collected evidence about how these probes perform in reporting action potential transmission as a function of their expression in the various compartments of the neuron. Voltage indicators and sensors differ in terms of speed and kinetics of depolarization and repolarization, sensitivity, colour and brightness, as well as of level of expression at the axonal terminal. These tools have helped to reveal the propagation of the electrical signal along the axon. The class of GEVIs that is more efficiently expressed at the axonal level includes microbial rhodopsin‐based indicators, such as QuasArs (Hochbaum et al., 2014), derived from the site‐directed mutagenesis of Arch voltage indicators and aimed at enhancing sensitivity to voltage changes, and FRET‐opsin indicators (Gong et al., 2014), which are based on matching an acceptor molecule (the opsin) with a donor one (fluorophores or synthetic dyes). A different and very effective approach exploited voltage sensitive phosphatases (VSPs); the most sensitive variants of these sensors, named ArchLight and Bongwoory, were obtained by fusing the Ciona intestinal VSP (CiVSP) to a mutated super‐ecliptic pHLuorin, whose fluorescence was found to heavily depend on the conformational changes of the VSP induced by membrane depolarization (Jin et al., 2012; Lee et al., 2017).

Thanks to the development of new spectral variants of these tools, it is now possible to simultaneously monitor different aspects of synaptic transmission, for example by expressing a far‐red rhodopsin based GEVI on the synaptic membrane, a red Ca^2+^ indicator in the presynaptic cytoplasm and Synapto‐phLuorin in the vesicle lumen. However, no VS has been specifically targeted to the synapse, which would be desirable for future investigations.

### 2.6 Sensing neurotransmitter release

Several synthetic and genetically encoded tools are available for directly sensing the presence of neurotransmitters both in vitro and in vivo. Such approach is very specific in terms of the signalling molecules involved in transmission. The toolkit of synthetic probes includes fluorescent false neurotransmitters (FFNs), which mimic the endogenous monoamines: FN200 is loaded and released specifically by vesicles containing the vesicular monoamine transporter 2 (VMAT2) (Pereira et al., 2016). This approach has proven successful in staining dopaminergic terminals in vitro, and the unloading process can be monitored to estimate the rate of neurotransmitter release, similarly to FM 1‐43 (Betz & Bewick, 1993). Another synthetic probe is the pH‐sensitive neurotransmitter binder ExoSensor517 (Klockow et al., 2013), whose fluorescence is increased only when it binds to glutamate in the acidic environment of the vesicle, so that also in this case, the destaining can be used to measure glutamate exocytosis. However, this probe is not interacting with any synaptic protein, and thus, synaptic specificity is not guaranteed. Other commendable seminal work in this field was aimed at developing hybrid sensors, based on synthetic molecules interacting with receptor proteins such as alpha‐amino‐3‐hydroxy‐5‐methyl‐4‐isoxazole‐propionic acid receptor (AMPAR) (Brun et al., 2012; Okubo et al., 2010) and GABA receptor (Masharina et al., 2012). Nevertheless, these sensors, even when based on synaptic receptors, mainly provide a readout of extrasynaptic concentrations of the neurotransmitter.

In the vast set of genetically encoded neurotransmitter sensors, the first to be developed and the most refined over the years is iGluSnFR (Marvin et al., 2013). This sensor is based on a bacterial periplasmic binding protein (PBP), the GltI from *E. coli*, which binds to glutamate, chimerized with circularly permutated GFP (cpGFP), so that conformational change of GltI after interaction with glutamate affects cpGFP fluorescence. The sensor was further improved and applied successfully in vivo with single synapse resolution (Helassa et al., 2018). A similar protein engineering strategy was successfully adopted using suited PBPs to detect other neurotransmitters, including GABA (iGABASnFR) (Marvin et al., 2019), ATP (iATPSnFR) (Lobas et al., 2019), serotonine (iSeroSnFR) (Unger et al., 2020) and acetylcholine (iAChSnFR) (Nichols et al., 2022). Importantly, most of these sensors have been proved effective in vivo using different model organisms and showed high sensitivity and selectivity, as well as fast kinetics (see Lin et al., 2021; Sabatini & Tian, 2020 for comprehensive reviews about genetically encoded neurotransmitter indicators). Nevertheless, because these PBP‐based neurotransmitter sensors do not exploit endogenous synaptic receptors as scaffolds, the signalling dynamics can be quite far from the physiological ones, and this can limit their informativeness. Furthermore, targeting of PBP‐based sensors at the synaptic compartment has not been achieved yet.

A different approach to construct neurotransmitter sensors was based, on the contrary, on the engineering of endogenous G‐coupled receptors (GPCRs). This appears as a more specific approach as the receptor, although being inert in terms of transmission, would show kinetics and affinity comparable with its naïve version, and its localization profile would reflect the physiological one. A dopamine (DA) sensors family named dLight1 was obtained by chimerization of DA metabotropic receptors (DRD1, DRD2 and DRD4) with cpGFP: As with PBP‐based sensors, the conformational change of the different GPCRs upon binding to DA leads to detectable changes in cpGFP fluorescence (Patriarchi et al., 2018). Interestingly, the authors show how dLight1 sensors provide robust reading of DA transients at dopaminergic terminals in the dorsal striatum of mice using two‐photon microscopy, demonstrating high temporal and spatial resolution, and the applicability of lower resolution imaging such as fibre‐photometry and epifluorescence microscopy of superficial layers of the motor cortex (Patriarchi et al., 2018). Almost parallel to this study, Sun and colleagues proposed a slightly different version of GPCR‐based sensors for DA, named GRAB_DA1_ and GRAB_DA1h_, and provided evidence for its efficacy both in vitro and in vivo, using similar techniques (two‐photon microscopy and fibre photometry) on mouse, drosophila and zebrafish (Sun et al., 2018). Improved (GRAB_DA2m_ and GRAB_DA2h_) and red‐shifted versions (rGRAB_DA1m_ and rGRAB_DA1h_) of GRAB_DA_ were recently developed (Sun et al., 2020). These results further testify the feasibility of such powerful design approach. As the matter of fact, in the last few years, other sensors were validated based on the same principle, allowing to detect, with high sensitivity, specificity and temporal resolution, the release of acetylcholine (GACh; this was obtained by fusing cpGFP with the muscarinic acetylcholine receptor, MR) (Jing et al., 2018), norepinephrine (GRAB_NE_; with a2AR receptor as scaffold) (Feng et al., 2019) and serotonin (5‐HT) (GRAB_5‐HT_; with 5‐HT2C receptor as scaffold) (Wan et al., 2021). All these studies provide convincing evidence about the applicability of these sensors in vivo, and their signal‐to‐noise ratio seems excellent, as well as the reported specificity for their neurotransmitters. Nevertheless, it is worth mentioning that none of these studies provided measurements of neurotransmitter dynamics with single synapse resolution in vivo. This should be taken into account for specific synaptic studies, as the readout might be in part due to extrasynaptic neurotransmitter release and spillover effects. Furthermore, some other points of attention can be raised for these GPCR‐based sensors: (i) The sensor is not totally inert, due to residual interaction with the target G protein; (ii) if strongly overexpressed, these sensors will not reflect the physiological distribution among cellular compartments, and they might act as a sink for the neurotransmitter, leading to depressed postsynaptic activation; (iii) GPCR moieties remain sensitive to several drugs, thus limiting many potential pharmacological investigations. Another genetically encoded sensor was constructed by fusing a synaptic protein, synaptophysin, to luciferase and the fluorescent protein mCherry: This sensor, named Syn‐ATP, allows to measure ATP release occurring at the presynaptic level following neural firing (Rangaraju et al., 2014). The use of luciferase requires the delivery of luciferin to the sample, which might affect synaptic transmission even if only marginally, as shown by the authors; in addition, no in vivo applications have been reported.

### 2.7 Sensors for labeling recently potentiated synapses

Subunits of AMPA receptors were fluorescently labelled to visualize the recent recruitment of glutamate receptors at postsynaptic terminals after long‐term potentiation (LTP) (Makino & Malinow, 2011). In their seminal work, the authors exploited the GluA1 and GluA2 AMPAR subunits fused to the Super Ecliptic pHluorin (SEP). By using these sensors, named SEP‐GluR1 and SEP‐GluR2, together with two‐photon microscopy in vivo, the authors showed that GluA1 is enriched at post‐synaptic spines after LTP, while GluA2 subunits are recruited after putative homeostatic plasticity remodelling of somatosensory cortex of mice (Makino & Malinow, 2011). As shown by the authors, incorporation of homomeric AMPA receptors formed by these recombinant subunits inevitably affects electrical responses by introducing rectification for positive membrane voltages. In addition, their expression must be conditionally regulated (the authors used a conditional Cre/loxP system) and should not exceed two days; otherwise, the signal appears as mainly related to the spine size rather than to recent AMPA incorporation (Makino & Malinow, 2011). A different approach was developed later by Hayashi‐Takagi et al. (2015) and exploited PSDΔ1.2, a mutant of PSD95 that is localized at postsynaptic terminal without competing for PDZ interaction, together with the dendritic targeting element (DTE) of *Arc*, to direct a fluorescent protein to postsynaptic dendritic spines that were recently significantly activated through NMDA receptors (Hayashi‐Takagi et al., 2015). The fluorescent protein selected was Venus, fused to a photoactivable small GTPase (PaRac1), whose function will be later described in this review. The resulting sensor was named AS‐PaRac1 (where ‘AS’ stands for ‘activated synapse’). Thanks to the NMDAR‐dependent targeting of this construct at synapses, the authors were able to label synapses that underwent an associative form of LTP in a recent timeframe (Hayashi‐Takagi et al., 2015). One limitation of the method resides in the specificity of the LTP induction mechanism. In addition, it is not clear to which extent NMDAR activations that are not able to drive LTP (or that even drive LTD) still produce detectable AS‐PaRac1 targeting, which would exert a confounding effect.

**FIGURE 1:**
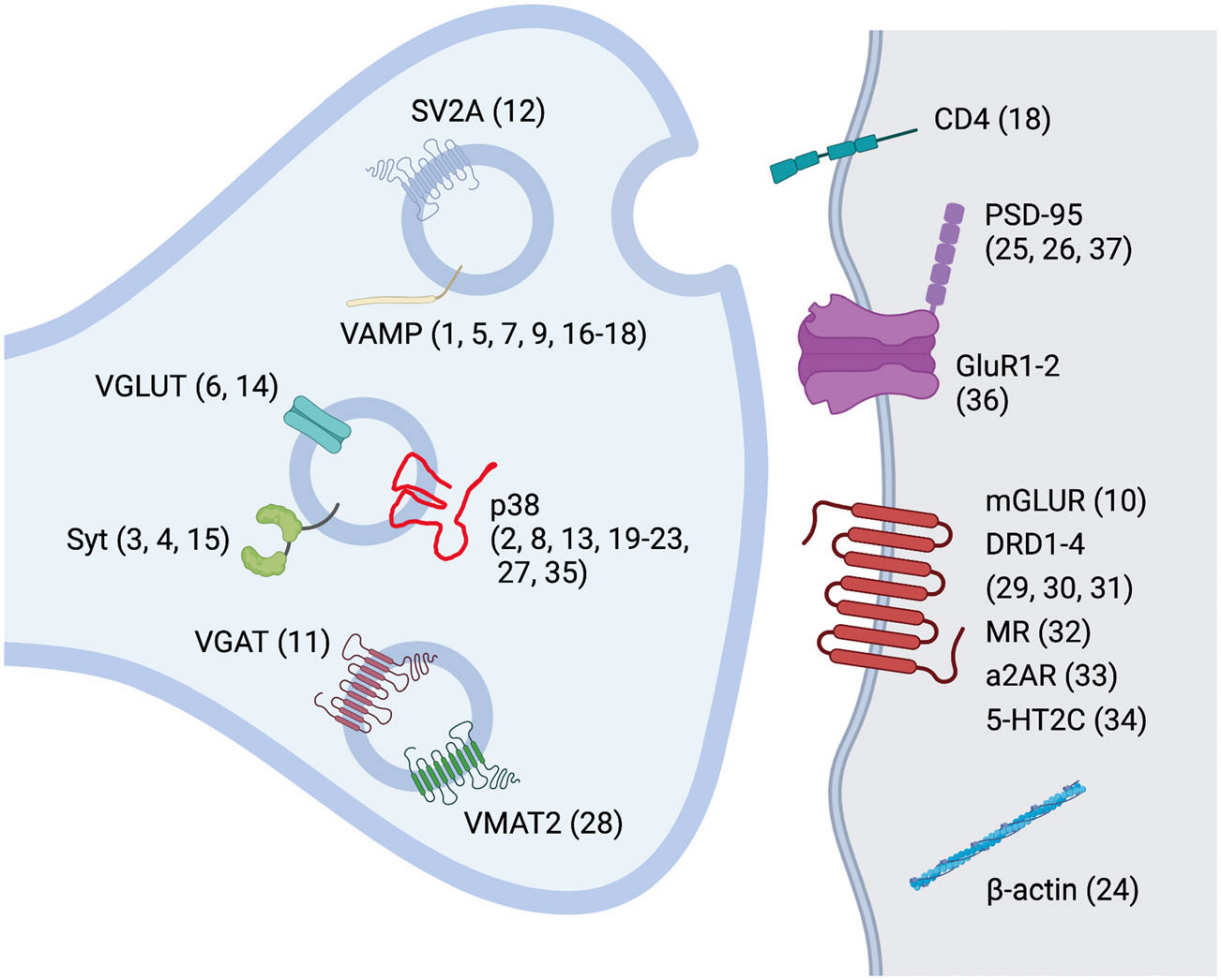
Synaptic proteins exploited for developing sensors (numbers in parentheses refer to Table 1, first column). Synaptobrevin (VAMP) is used by Synapto‐pHluorin (1), VAMP4‐pHluorin (5), syb2‐mOrange (7), VAMP2‐pHmScarlet (9), eGFP‐SynaptoZip (16), mCherry‐SynaptoZip (17) and (together with CD‐4) by Syb‐GRASP (18). Synaptophysin (p38) is used by SypHy (2), SypHTomato (8), pHoenix (13), SyGCaMP2 (19), SyGCaMP3 (20), SyGCaMP5G (21), SyGCaMP6 (22), SypHy‐RGECO (23), preSynTagMA (27) and Syn‐ATP (35). Synaptotagmin (syt, p65) is used by pHluorin‐syt‐IV (3), pHluorin‐syt‐1‐17 (4) and anti‐p65 (15). VGLUT is used by VGLUT1‐pHluorin (6) and VGLUT1‐mOr2 (14). VGAT is used by VGAT‐pHluorin (11). Synaptic vesicle protein 2A (SV2A) is used by SV2A‐pHluorin (12). β‐Actin is used by GCaMP2‐actin (24). PSD‐95 is used by PSD95‐GCaMP2 (25) and PSD‐95‐GCaMP5K (26), whereas its mutation PSDΔ1.2 is used by AS‐PaRac1 (37). VMAT2 is used by FN200 (28). GluR1 and GluR2 are used by SEP‐GluR1 and SEP‐GluR2 (36). As for G‐protein coupled receptors, mGLUR is used by pHluorin‐tagged mGluR7 (10); DRD1, DRD2 and DRD4 are used by dLight (29); DRD2 is used by GRAB_DA_ (30) and rGRAB_DA_ (31); MR is used by GACh (32); a2AR is used by GRAB_NE_ (33); and 5‐HT2C is used by GRAB_5‐HT_ (34).

Table 1 provides a selection of the sensors described in this section, along with their main features, limited to those that exploited a synaptic protein for their development, and Figure 1 depicts the synaptic location of the proteins involved in their operating principle.

**TABLE 1:** Methods for measuring synaptic communication based on synaptic proteins

	Name	Protein	Sensor	Principle	GE	Readout	In vitro	In vivo	Key limitations	Ref.
1	Synapto‐pHluorin	VAMP‐2	pHluorin (ecliptic GFP)	pH sensor	Yes	Exo‐endocytosis	Yes	No	Presynaptic only Low sensitivity Transient signal Low SNR Complex imaging in vivo	Miesenböck et al. (1998)
2	SypHy	p38	pHluorin (ecliptic GFP)	pH sensor	Yes	Exo‐endocytosis	Yes	No		Granseth et al. (2006)
3	pHluorin‐syt‐IV	syt 4	pHluorin (ecliptic GFP)	pH sensor	Yes	Exo‐endocytosis	Yes	No		Dean et al. (2009)
4	pHluorin‐syt‐1‐17	syt 1–7 syt 9–12, 17	pHluorin (ecliptic GFP)	pH sensor	Yes	Exo‐endocytosis	Yes	No		Dean, Liu, et al. (2012)
5	VAMP4‐pHluorin	VAMP‐4	pHluorin (ecliptic GFP)	pH sensor	Yes	Exo‐endocytosis	Yes	No		Nicholson‐Fish et al. (2016)
6	VGLUT1‐pHluorin	VGLUT1	pHluorin (ecliptic GFP)	pH sensor	Yes	Exo‐endocytosis	Yes	No		Voglmaier et al. (2006)
7	syb2‐mOrange	VAMP‐2	mOrange	pH sensor	Yes	Exo‐endocytosis	Yes	No		Ramirez et al. (2012)
8	SypHTomato	p38	pHTomato	pH sensor	Yes	Exo‐endocytosis	Yes	No		Li & Tsien (2012)
9	VAMP2‐pHmScarlet	VAMP‐2	pHmScarlet	pH sensor	Yes	Exo‐endocytosis	Yes	No		Liu et al. (2021)
10	pHluorin‐tagged mGluR7	mGLUR7	pHluorin (ecliptic GFP)	pH sensor	Yes	Exo‐endocytosis	Yes	No		Pelkey et al. (2007)
11	VGAT‐pHluorin	VGAT	pHluorin (ecliptic GFP)	pH sensor	Yes	Exo‐endocytosis	Yes	No		Santos et al. (2013)
12	SV2A–pHluorin	SV2A	pHluorin (ecliptic GFP)	pH sensor	Yes	Exo‐endocytosis	Yes	No		Zhang et al. (2015)
13	pHoenix	p38	pHluorin (ecliptic GFP)	pH sensor	Yes	Exo‐endocytosis	Yes	No		Rost et al. (2015)
14	VGLUT1‐mOr2	VGLUT1	mOr2	pH sensor	Yes	Exo‐endocytosis	Yes	No		Li (2011)
15	anti‐p65	syt (p65)	p65 antibody	vesicle tag	No	Exo‐endocytosis	Yes	No	Antibody delivery	Malgaroli et al. (1995)
16	eGFP‐SynaptoZip	VAMP‐2	ZIP/Synbond eGFP	vesicle tag	Yes	Exo‐endocytosis	Yes	Yes	Presynaptic only SB delivery Temporal dynamics	Ferro et al. (2017)
17	mCherry‐SynaptoZip	VAMP‐2	ZIP/Synbond mCherry	vesicle tag	Yes	Exo‐endocytosis	Yes	Yes		Lamanna et al. (2022)
18	Syb‐GRASP	VAMP‐2CD‐4	split‐GFP1‐10 split‐GFP11	synapse tag	Yes	Exo‐endocytosis	Yes	Yes	Sensor and vesicles sequestration No quantification Low fluorescence intensity	Macpherson et al. (2015)
19	SyGCaMP2	p38	GCaMP2	Ca^2+^ sensor	Yes	Synaptic activation	Yes	Yes	Early phase of synaptic activation No spontaneous transmission	Dreosti et al. (2009)
20	SyGCaMP3	p38	GCaMP3	Ca^2+^ sensor	Yes	Synaptic activation	Yes	Yes		Li (2011)
21	SyGCaMP5G	p38	GCaMP5G	Ca^2+^ sensor	Yes	Synaptic activation	Yes	Yes		Akerboom et al. (2012)
22	SyGCaMP6	p38	GCaMP6	Ca^2+^ sensor	Yes	Synaptic activation	Yes	No		Mahn et al. (2016)
23	SypHy‐RGECO	p38	pHluorin (ecliptic GFP) RGECO1	pH sensor Ca^2+^ sensor	Yes	Synaptic activation and exocytosis	Yes	No		Jackson & Burrone (2016)
24	GCaMP2‐actin	β‐Actin	GCaMP2	Ca^2+^ sensor	Yes	Synaptic activation	Yes	No		Mao et al. (2008)
25	PSD95‐GCaMP2	PSD‐95	GCaMP2	Ca^2+^ sensor	Yes	Synaptic activation	Yes	No		Mao et al. (2008)
26	PSD‐95‐GCaMP5K	PSD‐95	GCaMP5K	Ca^2+^ sensor	Yes	Synaptic activation	Yes	No		Leitz & Kavalali (2014)
27	preSynTagMA	p38	CaMPARI‐2	Ca^2+^ sensor	Yes	Synaptic activation	Yes	Yes		Perez‐Alvarez et al. (2020)
28	FN200	VMAT2	FN200	False neuro‐transmitter	No	Dopaminergic exo‐endocytosis	Yes	No	FN200 delivery Low sensitivity Transient signal	Pereira et al. (2016)	
29	dLight1	DRD1‐2,4	cpGFP	DA sensor	Yes	DA release	Yes	Yes	Low spatial resolution Extrasynaptic release Residual GPCR activity Buffering action sensitivity to drugs Non‐physiological distribution	Patriarchi et al. (2018)	
30	GRAB_DA_	DRD2	cpGFP	DA sensor	Yes	DA release	Yes	Yes		Sun et al. (2018)	
31	rGRAB_DA_	DRD2	cpGFP	DA sensor	Yes	DA release	Yes	Yes		Sun et al. (2020)	
32	GACh	MR	cpGFP	ACh sensor	Yes	ACh release	Yes	Yes		Jing et al. (2018)	
33	GRAB_NE_	a2AR	cpGFP	NE sensor	Yes	NE release	Yes	Yes		Feng et al. (2019)	
34	GRAB_5‐HT_	5‐HT2C	cpGFP	5‐HT sensor	Yes	5‐HT release	Yes	Yes		Wan et al. (2021)	
35	Syn‐ATP	p38	luciferase and mCherry	ATP sensor	Yes	ATP release	Yes	No	Luciferin delivery Altered presynaptic function Only evoked release	Rangaraju et al. (2014)
36	SEP‐GluR1 SEP‐GluR2	GluR1 GluR2	Super ecliptic pHluorin (SEP)	AMPAR tag	Yes	AMPA recruitment	Yes	Yes	Conditional expression Limited time window Altered electrical responses	Makino & Malinow (2011)	
37	AS‐PaRac1	PSDΔ1.2	Venus Rac1 *Arc* DTE	Postsynaptic tag	Yes	synaptic LTP	Yes	Yes	NMDA‐dependent LTP only LTP induction only	Hayashi‐Takagi et al. (2015)	

## 3 EXPLOITING SYNAPTIC PROTEINS TO CONTROL SYNAPTIC COMMUNICATION

A complete methodological approach would require not only to gather observations on the physiological functioning of synaptic boutons but also to perform loss‐ and gain‐of‐function experiments with high genotype selectivity and temporal resolution. Nowadays, this requirement is largely met for controlling neuronal electrical activity thanks to the rapid evolution of optogenetics (Fenno et al., 2011). Optogenetics combines optical control and genetic targeting of specific neurons or proteins. In the past decades, optogenetic techniques have been widely used for accurate spatiotemporal regulation of cellular functions; their non‐invasiveness, reversibility and fast response time provide them with great potential for the study of many different biological systems (Deisseroth et al., 2006). In chemogenetics, the genetic strategy is exploited to introduce a drug‐activable receptor in lieu of a photoactivable channel. If optogenetics enables precise temporal control of neuronal firing, chemogenetics modulates it for a long period of time with a single administration of a designer drug (Vlasov et al., 2018). As for the specific modulation of synaptic communication, some developments have been made, but the toolkit remains quite limited.

### 3.1 Disruption of synaptic exocytosis based on SNAREs cleavage

A first attempt was aimed at inhibiting synaptic exocytosis using a recombinant *Chlostridium tetani* toxin that can be selectively internalized by a genetically encoded human interleukin‐2 receptor (IL‐2Rα) subunit (Kobayashi et al., 2008). The authors engineered an alternative version of the tetanus toxin (ITet) by fusing its light chain (TeTx‐L) to the variable region of a monoclonal antibody against IL‐2Rα. This approach has proven effective in inhibiting synaptic transmission in specific regions thanks to the promoter‐driven expression of the IL‐2Rα receptor in transgenic mice, whose synapses were able to internalize the ‘immunotoxin’ ITet causing proteolytic VAMP‐2 cleavage (Kobayashi et al., 2008). However, some limitations of this method are expected in terms of spatial and temporal resolution, as the purified recombinant toxin must be delivered to the tissue and the duration of the effect, without dialysis washout, would be related to the half‐life of the toxin, which is equal to about 5‐6 days for both types of chains (Habig et al., 1986). Such timing seems in line with the results shown by the authors for in vivo stereotaxic injection of ITet, whose silencing effect reaches its peak at Day 4 post injection and is nulled at Day 10; although the authors also tested microdialysis, which is expected to facilitate toxin washout, the evaluated time points cannot support such view (Kobayashi et al., 2008).

### 3.2 Using light to control presynaptic activity

A more recent approach to inhibit synaptic exocytosis, developed by Lin and colleagues (Lin et al., 2013), was based on chromophore‐assisted light inactivation (CALI), a phenomenon that allows to inactivate proteins thanks to the generation of reactive oxygen species by miniSOG. This tool was named InSynC and consists of the fusion of miniSOG to either VAMP‐2 or synaptophysin. When expressed in cultured neurons, in organotypic slices and in neurons from *C. elegans*, InSynC can strongly inhibit synaptic transmission upon blue light stimulation of the sample, and such inhibition lasts more than 1 h (Lin et al., 2013). Although this approach appears superior in terms of spatial and temporal resolution when compared with the one based on VAMP‐2 cleavage (see previous paragraph), some limitations still exist: The inhibited state is not complete and cannot be manually reversed. In addition, a light‐power vs. inhibition relationship was not provided by the authors. An alternative approach for synaptic inhibition, successfully applied in *C. elegans*, involves the replacement of endogenous synaptotagmin with the same protein fused to a photosensitive degron (Hermann et al., 2015). Upon light illumination, the degron degrades synaptotagmin, thus compromising exocytosis (Hermann et al., 2015). Unfortunately, degron‐mediated protein degradation will not be complete, so that exocytosis, also in this case, will not be totally lost; however, this method enables precise action, thanks to its targeting at the synaptic site. In a recent study by Won et al. (2021), VAMP‐2 was conjugated to LARIAT, an optogenetic tool composed of the light‐responsive CRY2 and its target protein CIB1. The capability of this system, named Opto‐vTrap, is to transiently trap vesicles to block exocytosis: Upon blue light stimulation, LARIAT goes through homomeric oligomerization and sequesters transmitter‐containing vesicles with spontaneous recovery after 30 min from light removal (Won et al., 2021). Although a relatively low level of blockage of memory retrieval was observed with in vivo experiments, it demonstrated a fast recovery time (15 min to fully reverse sequestration), voltage and calcium independence and high inhibition efficiency.

An optogenetic construct that was already mentioned in the previous section as a variant of sypHy was obtained by inserting both archaerhodopsin‐3 (Arch3), a light‐activated outward proton pump commonly used to inhibit neurons, and pHluorin, between the third and fourth transmembrane domains of synaptophysin. Through this system, named pHoenix, accumulation of protons within the synaptic vesicle can be promoted, which, in turn, drives the vesicular loading of neurotransmitters (Rost et al., 2015). This action was shown to enhance transmission, but this effect does not seem to be reversible.

Additional tools based on optogenetic proteins targeted at synapses have been developed in recent years: An example is SynaptoPAC (Oldani et al., 2020) that was aimed at controlling the induction and expression of presynaptic short‐ and long‐term potentiation. This construct was obtained by fusing bPAC, the *Beggiatoa* photoactivated adenylyl cyclase, to the synaptic vesicle protein synaptophysin, thus enabling light‐induced cAMP production localized at axonal terminals. Such cAMP elevation was found to induce presynaptic potentiation only at granule cell terminals, leaving other cell types unaffected. An optogenetic construct named mGluR2‐PA‐ChR2 has later been engineered by Hamada et al. (2021) by fusing ChR2 to the C‐terminal domain of the metabotropic glutamate receptor 2 (mGluR2) along with a proteolytic motif and an axon‐targeting element (Hamada et al., 2021). This tool offers a way to limit optogenetic stimulation to the axon. Although the targeting of this construct to the axon was shown effective, it is known that mGlur2 resides at both pre‐ and postsynaptic sites (Petralia et al., 1996), as well as on the cytoplasmic membrane of astrocytes (Jin et al., 2017).

### 3.3 Targeting opsins at the postsynaptic terminal

Other genetically encoded tools were designed to target microbial opsins to the postsynaptic compartment, in order to modulate postsynaptic electrical activation. A seminal example is provided by the protein engineered by Gradinaru et al. (2007), obtained by adding the sequence ETQV, an aminoacidic sequence known to bind the PDZ‐domain of PSD‐95, to channelrohdopsin‐2 (ChR2) (Gradinaru et al., 2007). This resulted in concentrated expression of ChR2 at postsynaptic sites, albeit the reliability and localization of photocurrents induced using this probe were not characterized by the authors. A further attempt to limit the expression of ChR2 to the dendritic tree is described in the study by Smirnova et al. (2019), where the PSD protein Homer1 was used as an anchoring motif for ChR2, aimed at localizing the opsin at the peripheral districts of primary cultured neurons (Smirnova et al., 2019). The authors showed only a modest (but detectable) localization of this construct at the soma, although its presence at this compartment and at different distances on the dendritic arbor was not evaluated when higher levels of construct expression were promoted. Another recent tool, named interluminescent optical synapse, allows to modulate transmission at synapses that express the blue‐light emitting luciferase (sbGluc) in presynaptic vesicles and an opsin postsynaptically. The authors fused sbGluc to the human proopiomelanocortin pro‐peptide (hPOMC1‐26) to target synaptic vesicles and expressed either ChR2 or hGtACR2 in postsynaptic neurons, to obtain either excitation or inhibition of postsynaptic terminals, respectively. Only when luciferin coelenterazine (CTZ) is provided to the tissue and vesicles fuse to the presynaptic membrane, the bioluminescence generated by released sbGluc activates its partner opsin and either excites or inhibits the postsynaptic membrane in an activity‐dependent manner (Prakash et al., 2022). When the tool is used in vivo, the administration of CTZ via craniotomy proved to be complex. In fact, the diffusibility of CTZ likely limits the applicability of the system to the most superficial cortical layers, and its diffusion dynamics likely affect the time course of the signal, raising doubts as to whether the system is really advantageous compared with the excitation/inhibition of neural circuits obtained by classical optogenetics approaches.

### 3.4 Chemogenetic and optogenetic control of metabotropic pathways

A groundbreaking innovation for the in vivo control of neurotransmission is represented by the engineering of the so‐called Designer Receptors Exclusively Activated by Designer Drug (DREADDs). These are modified GPCRs that can be activated only by the exogenous inert molecule clozapine‐N‐oxide (CNO), thus allowing control of neuronal activity with genotypic selectivity and easy delivery of the drug (Alexander et al., 2009). This approach was later exploited to control neuronal communication presynaptically using the Gi‐protein‐coupled receptor hM4Di (Stachniak et al., 2014). The authors showed that hM4Di activated by CNO was able to inhibit synaptic transmission both in vitro and in vivo without altering excitability of the neuron at the soma and over the axon. Furthermore, the hM4D^nrxn^ variant was developed by addition of an amino acid sequence derived from neurexin‐1 at the intracellular end and shown to obtain the same degree of synaptic silencing with even less hyperpolarization of the cell membrane (Stachniak et al., 2014). The main limitation to the use of hM4D^nrxn^ to selectively silence synaptic projections relates to the need for administering intracranially the solution containing CNO, which limits the temporal dynamics of inhibition, also requiring the use of positive pressure, which might result in mechanical stimulation of the tissue. In addition, such injections turned out to be unsuitable for electrophysiological recordings because of the resulting tissue movement, as well as for behavioural experiments, as reported by the authors (Stachniak et al., 2014). Using a similar approach, Copits and colleagues exploited the lamprey parapinopsin (POO), a photoswitchable opsin that couples to G_i/o_ GPCRs, as a presynaptic silencer (Copits et al., 2021). POO can be activated by pulsed blue light and switched off using green/amber light, thus rapidly and reversibly controlling inhibitory G‐protein pathways. The authors were able to inhibit in vivo the release of glutamate, GABA and DA at presynaptic terminals, thus altering reward behaviours (Copits et al., 2021). Here, some of the caveats just described for the previous probe still apply. In addition, the photo‐switching of POO might act as a high‐pass filter becoming less effective during bursts of activity; for this reason, a more detailed understanding of the conformational dynamics of PPO would be beneficial for its use in this application.

### 3.5 Controlling synaptic plasticity with optogenetics

The first photoactivable construct developed to structurally modify synapses was already introduced above: AS‐PaRac1 (Hayashi‐Takagi et al., 2015). As the matter of fact, this probe has been proven able not only to localize at postsynaptic sites that have been recently potentiated (as described in the previous section) but also to produce dendritic spine shrinking upon light stimulation, thanks to the enzymatic activity of the embodied small GTPase Rac1. Interestingly, this effect paralleled a loss in functional transmission at those spines, as evidenced by depressed calcium imaging transients, thus supporting the effectiveness of this approach in reverting recently occurred postsynaptic potentiation. The authors also showed that motor skills recently acquired by mice on a rotarod can be selectively erased by low frequency, blue‐light illumination of AS‐PaRac1 expressing spines in motor cortex layer I (Hayashi‐Takagi et al., 2015).

Goto and colleagues (Goto et al., 2021) exploited the same CALI principle described above and fused cofilin, an actin‐depolymerizing protein, to the genetically encoded photosensitizer protein SuperNova (SN) (Takemoto et al., 2013). Upon illumination, SN inactivates cofilin, leading to the destabilization of the cofilactin structures within the dendritic spines of hippocampal neurons. With the use of this approach, the authors were able to revert structural LTP for investigating its involvement in the different temporal phases of memory engram formation, including consolidation during sleep (Goto et al., 2021). Although the results shown for their validation are very promising, the effect of both AS‐PaRac1 and SN‐cofilin is mainly morphological and only indirectly functional, beside leading to a very specific form of synaptic plastic changes. In addition, the former tool is effective only for recently potentiated synapses.

Another really versatile approach to the optogenetic manipulation of synaptic proteins was developed by Sinnen and colleagues (Sinnen et al., 2017) and is based on a dimeric system derived from *Arabidopsis thaliana*. This system is composed of the photoreceptor cryptochrome 2 (CRY2) that interacts with its counterpart CIB1 upon light illumination. CRY2 was fused to structural postsynaptic proteins, namely, homer1c, PSD95 and a genetically encoded antibody against PSD95, whereas CIB1 was fused to the AMPA receptor subunit GluA1. The authors showed that, upon blue‐light illumination, AMPA receptors are rapidly recruited to the postsynaptic membrane of spines, concentrated at the PSDs, and that this condition is then spontaneously reverted, albeit this might require tents of minutes depending on the stimulus duration. The authors reported 90% dissociation after 12 min from a single exposure, but the kinetics for the GluA1 recruitment experiments seems much slower. As for the functional effects of GluA1 concentration at PSDs, extended electrophysiological analyses showed that quantal frequency is increased, whereas quantal amplitude is unaffected, suggesting activation of previously silent synapses (Sinnen et al., 2017). This approach appears very promising for future applications based on the manipulation of molecular elements of the postsynaptic compartment.

Opposed to the previous study, a new optogenetic tool named PhotonSABER was developed by Kakegawa et al. (2018), allowing precise temporal and spatial control of AMPA receptor endocytosis at active synapses (Kakegawa et al., 2018). In order to inhibit it, the authors expressed an engineered photosensitive proton (H+) pump (ASR^D217E^) in endosomes by fusing it to the C‐terminus of chloride channel protein 5. When stimulated with light (green‐yellow light being the most effective), PhotonSABER allows to reversibly increase endosomal pH in a rapid fashion, thus inhibiting endocytosis and synaptic AMPA receptors removal. A point of concern may relate to the unwanted secondary effects that can be introduced by endocytosis blockade. Interestingly, this approach was used to block LTD involving Purkinje cells both in vitro and in vivo (Alexander et al., 2009). This application shows that having control of intracellular processes in the pre‐ or postsynaptic compartments might allow not only to drive or to inhibit synaptic communication but also to tune synaptic efficacy in the brain, with important behavioural consequences. Such result might contribute to shed light on the still marginally understood link between synaptic plasticity and animal behaviour.

**FIGURE 2:**
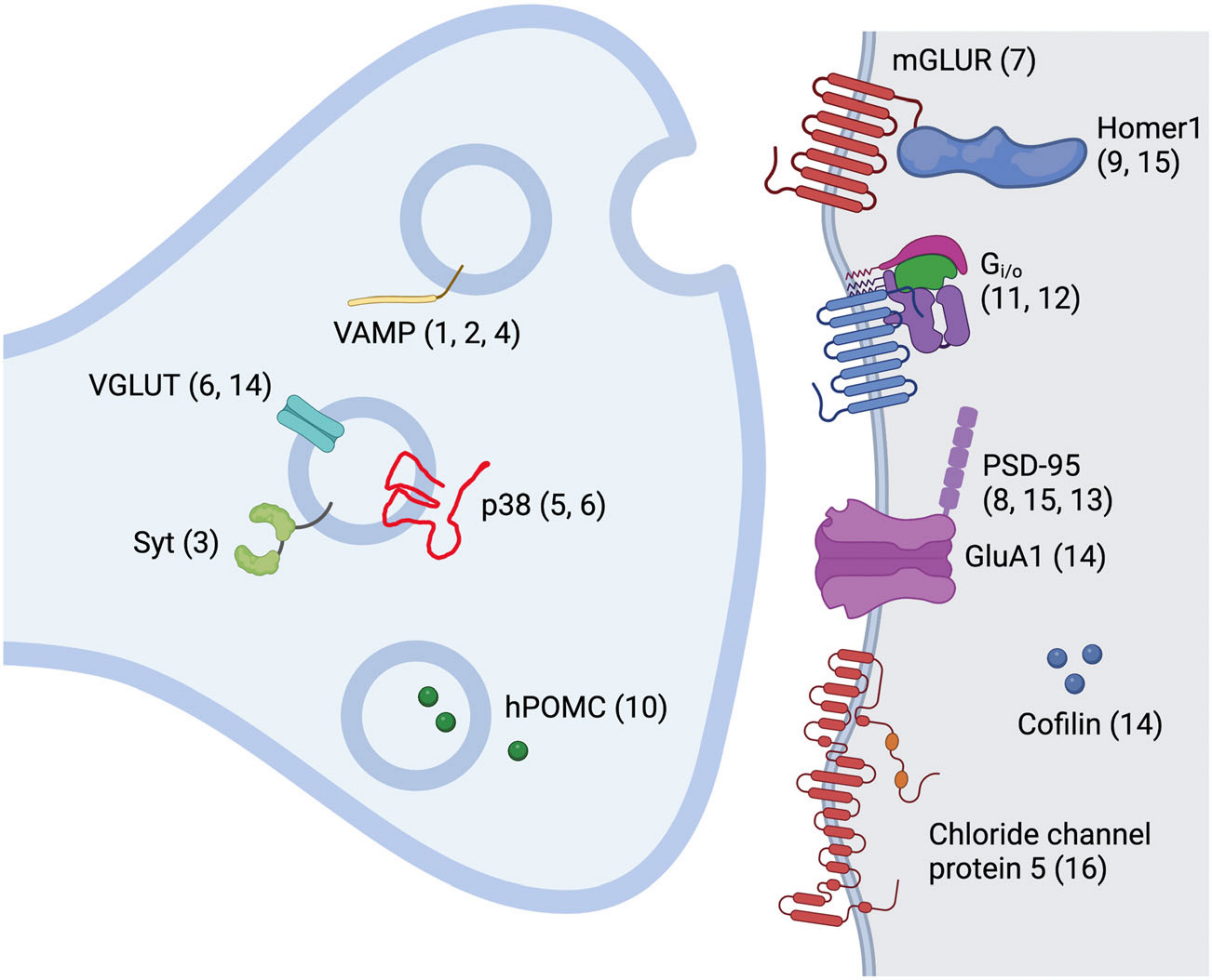
Synaptic proteins exploited for developing synaptic actuators (numbers in parentheses refer to Table 2, first column). VAMP is used by ITet (1), InSynC (2) and Opto‐vTrap (4). Cofilin is used by SN‐cofilin (14). Syt is used by SNT‐1::tagRFP::psd (3). P38 is used by pHoenix (5) and SynaptoPAC (6). mGluR is used by mGluR2‐PA‐ChR2 (7). Homer1 is used by pCAG‐ChR2‐Venus‐Homer1 (9) and CRY2‐GFP‐homer1c (15). hPOMC1‐26 is used by Interluminescent optical synapse (10). G_i_ with or without neurexin‐1 is used by hM4Di and hM4Di^nrxn^, respectively (11). G_i/o_ is used by POO (12). PSD‐95 is used by ChR2‐EYFP‐ETQV (8), CIB‐mCh and CIB‐mCh‐CaMKII (15), the latter using also GluA1. A mutated form of PSD‐95 (PSDΔ1.2) is also used by AS‐PaRac1 (13). Chloride channel protein 5 is used by PhotonSABER (16).

As above, the tools described in this section, whose development and functioning were based on specific synaptic proteins, are listed in Table 2, with Figure 2 showing the location of the involved proteins in the synaptic compartment.

**TABLE 2:** Methods for controlling synaptic communication based on synaptic proteins

	Name	Protein	Actuator	Principle	GE	Effect	In vitro	In vivo	Key limitations	Ref.
1	ITet	VAMP‐2	rTeTx‐L IL‐2Rα	VAMP‐2 cleavage	Yes	Inhibition of exocytosis	Yes	No	Low spatial and temporal resolution Toxin degradation Toxin delivery	Kobayashi et al. (2008)
2	InSynC	VAMP‐2 p38	miniSOG	CALI	Yes	Inhibition of exocytosis	Yes	No	Partial inhibition No control on recovery Light vs. inhibition unknown	Lin et al. (2013)
3	SNT‐1::tagRFP::psd	syt	psd	Protein photodegradation	Yes	Inhibition of exocytosis	Yes	Yes	Partial inhibition Dependence on resynthesis	Hermann et al. (2015)
4	Opto‐vTrap	VAMP‐2	CRY2/CIB1	Light‐controlled oligomerization	Yes	Vesicle sequestration	Yes	Yes	Low in vivo efficacy	Won et al. (2021)
5	pHoenix	p38	Arch3	Acidification of vesicle	Yes	Neurotransmitter loading	Yes	No	Irreversible	Rost et al. (2015)
6	SynaptoPAC	p38	bPAC	Light‐controlled cAMP synthesis	Yes	Presynaptic potentiation	Yes	No	Cell‐type specific Presynaptic only	Oldani et al. (2020)
7	mGluR2‐PA‐ChR2	mGluR2	ChR2	Light‐controlled excitation	Yes	Generation of antidromic spikes	Yes	Yes	Specificity limited by mGluR2 localization	Hamada et al. (2021)
8	ChR2‐EYFP‐ETQV	PSD‐95	ChR2	Light‐controlled excitation	Yes	Electrical activation of spines	Yes	No	Unknown reliability and localization of photocurrents	Gradinaru et al. (2007)
9	pCAG‐ChR2‐Venus‐Homer1	Homer1	ChR2	Light‐controlled excitation	Yes	Dendritic depolarization	Yes	No	Not complete confinement of expression	Smirnova et al. (2019)
10	Interluminescent optical synapse	hPOMC1‐26	sbGLuc ChR2 hGtACR2	Bioluminescence‐controlled excitation/inhibition	Yes	Synaptic transmission modulation	Yes	Yes	External delivery of luciferin coelenterazine	Prakash et al. (2022)
11	hM4Di/hM4Di^nrxn^	G_i_/neurexin‐1	hM4Di	Control of inhibitory metabotropic pathway	Yes	Inhibition of presynaptic transmission	Yes	Yes	External delivery of CNO	Stachniak et al. (2014)
12	POO	G_i/o_	POO	Control of inhibitory metabotropic pathway	Yes	Inhibition of presynaptic transmission	Yes	Yes	Temporal dynamics of PPO photo‐switching	Copits et al. (2021)
13	AS‐PaRac1	PSDΔ1.2	Venus Rac1 *Arc* DTE	Light‐controlled GTPase activity	Yes	Dendritic spine shrinking	Yes	Yes	Morphological plasticity Recently potentiated synapses	Hayashi‐Takagi et al. (2015)
14	SN‐cofilin	Cofilin	SuperNova	CALI	Yes	Selective erasure of LTP	Yes	No	Morphological plasticity	Goto et al. (2021)
15	CIB‐mCh CRY2‐GFP‐homer1c CIB‐mCh‐CaMKII	PSD‐95 homer1c GluA1	CRY2/CIB1	Light‐controlled dimerization	Yes	Recruitment of GluA1 subunits at PSDs	Yes	No	Slow reversal kinetics	Sinnen et al. (2017)
16	PhotonSABER	Chloride channel protein 5	ASR^D217E^	Light‐control of endosomal pH	Yes	Inhibition of AMPA removal	Yes	Yes	Specific form of LTD indirect effects unknown	Kakegawa et al. (2018)

## 4 LIMITATIONS, CAVEATS, AND FUTURE DEVELOPMENTS

The synaptic methods introduced above have been already applied in specific experimental settings either for validation purposes or to investigate different aspects of synaptic functioning. All these methods are promising, but several limitations still exist. Concerning the labelling of synaptic activation, as it can be appreciated by looking at Table 1 (column 9), in vivo applicability is lacking in most cases. This is a major concern, because it is now well established that the functional properties of synapses can be very different among neuronal cultures, acute slice preparations and the living brain (Borst, 2010). In addition, the activity of a synaptic bouton is at least in part dependent on the one of other millions belonging to the same pathway, and the latter is likely involved in a complex neural circuit, as in the thalamic‐cortico‐thalamic loop of basal ganglia (Sesack & Grace, 2010).

As described above, some exceptions exist: Calcium sensors localized at the presynaptic bouton such as SyGCaMP2 (Dreosti et al., 2009) can be reliably imaged in vivo with high resolution using two‐photon microscopy, albeit the live imaging requirement limits animal's behaviour and the depth of analysis. To solve this issue, recent approaches such as SynaptoZip (Ferro et al., 2017; Lamanna et al., 2022) and preSynTagMA (Perez‐Alvarez et al., 2020) provide a temporal integration of activity labelling to postpone the quantification analysis. Hypothetically, such capability might be extended also to other sensors; for example, those dependent on vesicular pH provided the availability of variants able to retain the generated signal. In addition, GECIs fused to non‐specific synaptic proteins have not been analysed here, but they can represent a very valuable resource for studying the dynamics of organelles such as the mitochondrion or the ER in the synaptic compartment, at least when imaging can be spatially confined to the latter (Suzuki et al., 2014; Wu et al., 2014).

As it clearly emerges by comparing the length of Tables 1 and 2, a wide gap in the availability of sensors and actuators based on synaptic proteins exists. This is likely related to the fact that, in the group of synaptic actuators, the optogenetic approach—which is relatively recent—is the most frequent. This is not surprising, given the large number of new variants of optogenetic switches that are now available (comprehensive information can be found at optobase.org, Kolar et al., 2018). Interestingly, most of these optogenetic tools remain to be tested as synaptic actuators. For example, the light‐oxygen‐voltage (LOV) sensing domain of *Avena sativa* has been successfully applied in several cases to drive molecular interactions and appears suited to control the interaction of synaptic proteins, such as the SNAREs, similarly to CALI (Lin et al., 2013). Furthermore, light‐based control has huge advantages, including high temporal resolution and minimal invasiveness, if compared with other modulation principles such as chemical or electrical stimulation. Hence, we can expect that the gap will be filled soon. It is worth noting that, at least for now, inhibiting exocytosis using light appears easier than evoking it. That said, different approaches should be evaluated in the future, also considering the current paucity of available methods. For example, recent studies showed the potential of magnetogenetics, an approach based on proteins acting as either magneto‐thermal or magneto‐mechanical transducers, that has been proven effective to non‐invasively stimulate neurons (Nimpf & Keays, 2017). Another example comes from the use of nanoparticles: Gold nanorods can be used to produce localized heating of neurons using near‐infrared light stimulation (Yong et al., 2014). Both these techniques might be theoretically applied also to the synaptic compartment.

Concerning the artificial manipulation of synaptic molecular composition, one of the most innovative approaches reported above relates to the insertion and/or removal of receptor channels in the postsynaptic membrane (Sinnen et al., 2017). Since the modification of synaptic receptor composition lies at the base of synaptic plasticity, this undoubtedly represents a promising tool to investigate the relationship between the expression of plasticity at defined neural circuits and animal behaviour, which is relevant in the etiopathology and treatment of many psychiatric disorders (Belujon & Grace, 2014; Ferro et al., 2021; Lamanna et al., 2020, 2022).

Unfortunately, the possibility to induce functional presynaptic changes, often required to express synaptic plasticity (Malgaroli et al., 1995), is still missing. These changes might artificially be mimicked by inducing the mobilization and/or recruitment of vesicles to the presynaptic AZ, or by inserting/removing voltage‐dependent calcium channels (VDCCs), although the effectiveness of such manipulations in modulating synaptic strength should still be established. More futuristic avenues relate to the *de novo* formation of synaptic contacts and axonal growth guidance, which would allow to reproduce and investigate realistic neural circuits in vitro with the help of novel techniques for neuronal culture patterning (Schulte et al., 2016, 2018).

The future availability of bidirectional genetically encoded synaptic strength modulators will also contribute to investigate a hot topic in neuroscience of learning and memory, which is the synaptic bases of memory engrams. Indeed, recent brilliant approaches allowed to identify and manipulate memory engrams at neuronal level (see Tonegawa et al., 2015 for a review), and some of the studies described here already succeeded in selectively erasing memories traces at synaptic level (Goto et al., 2021; Hayashi‐Takagi et al., 2015). Nevertheless, the artificial formation of cellular engrams encoding realistic memories by means of manipulations at the synaptic level has not been achieved yet and would represent a big step forward in the field.

## 5 CONCLUSION

In this review, we summarized the currently available methods for sensing or modulating synaptic activity and provided a selection of those based on modifications of specific synaptic proteins, residing either in the pre‐ or postsynaptic compartment. Although there are some limitations to the applicability of these methods, especially to the in vivo context, the overall picture is really promising because the available toolkit has never been so extended. The hope is that in the close future, some of these methods will be further implemented to obtain a functional synaptic readout from the entire brain. Importantly, many proofs‐of‐concept have been published in the last few years, suggesting totally new routes for obtaining more powerful approaches. In addition, the development of new genetically encoded synaptic actuators could allow to control the activity and the functional properties of synapses in vivo, a result that would help unveiling major questions in neuroscience, also extending the potential of neuromodulation for the effective treatment of mental disorders.

## CONFLICT OF INTEREST

The authors declare no competing financial interest.

## AUTHOR CONTRIBUTIONS

J.L., M.F., S. S and A.M. wrote and edited the manuscript.

6

### PEER REVIEW

The peer review history for this article is available at https://publons.com/publon/10.1111/ejn.15848.

## Data Availability

Data sharing is not applicable to this article as no new data were created or analyzed in this study.
